# Trends in patient‐reported outcome use in early phase dose‐finding oncology trials – an analysis of ClinicalTrials.gov

**DOI:** 10.1002/cam4.4307

**Published:** 2021-10-22

**Authors:** Julia Lai‐Kwon, Zhulin Yin, Anna Minchom, Christina Yap

**Affiliations:** ^1^ Drug Development Unit The Institute of Cancer Research and Royal Marsden Hospital London UK; ^2^ Clinical Trials and Statistics Unit The Institute of Cancer Research Sutton UK

**Keywords:** clinical trials, drug development, oncology, patient‐reported outcomes, phase 1, quality of life

## Abstract

**Background:**

Patient‐reported adverse events (AEs) may be a useful adjunct to clinician‐assessed AEs for assessing tolerability in early phase, dose‐finding oncology trials (DFOTs). We reviewed DFOTs on ClinicalTrials.gov to describe trends in patient‐reported outcome (PRO) use.

**Methods:**

DFOTs commencing 01 January 2007 – 20 January 2020 with ‘PROs’ or ‘quality of life’ as an outcome were extracted and inclusion criteria confirmed. Study and PRO characteristics were extracted. Completed trials that reported PRO outcomes and published manuscripts on ClinicalTrials.gov were identified, and PRO reporting details were extracted.

**Results:**

5.3% (548/10 372) DFOTs included PROs as an outcome. 231 (42.2%) were eligible: adult (224, 97%), solid tumour (175, 75.8%), and seamless phase 1/2 (108, 46.8%). PRO endpoints were identified in more trials (2.3 increase/year, 95% CI: 1.6–2.9) from an increasing variety of countries (0.7/year) (95% CI: 0.4–0.9) over time. PROs were typically secondary endpoints (207, 89.6%). 15/77 (19.5%) completed trials reported results on the ClinicalTrials.gov results database, and of those eight included their PRO results. Eighteen trials had published manuscripts available on ClinicalTrials.gov. Three (16.7%) used PROs to confirm the maximum tolerated dose. No trials identified who completed the PROs or how PROs were collected.

**Conclusions:**

PRO use in DFOT has increased but remains limited. Future work should explore the role of PROs in DFOT and determine what guidelines are needed to standardise PRO use.

## Graphical abstract

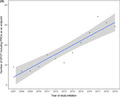

## 1 BACKGROUND

The primary purpose of early phase (phase 1 and phase 1/2) oncology trials is to establish the safety and tolerability of novel anti‐cancer agents. Clinician‐assessed adverse event (AE) reporting using the Common Terminology Criteria for Adverse Events (CTCAE) is critical for describing a drug's safety profile. CTCAE data, in conjunction with data on dose modifications, discontinuations and hospitalisations, is then used to select tolerable doses and regimens, including the maximum tolerated dose (MTD) and recommended phase 2 dose (RP2D).

However, clinician‐assessed AE reporting has limitations. Firstly, it may miss up to half of symptomatic AEs compared with patient self‐reports.^1^, ^2^, ^3^, ^4^, ^5^ Some AEs may be difficult to observe (e.g., fatigue) and not adequately characterised. This may under‐estimate toxicities and result in suboptimal toxicity management. Secondly, the patient's perspective on AEs is noticeably absent in the selection of tolerable doses and regimens.

Changes in the types of therapies in early phase oncology trials can also produce challenges in defining tolerable doses. Immunotherapy and targeted therapy may not produce toxicities in a dose‐dependent manner. Toxicities may be longer in duration or occur later than the traditional dose‐limiting toxicity (DLT) period, which is often defined as the first cycle of treatment.^6^, ^7^ Low‐grade toxicities experienced over the medium term to long term could reduce a drug's tolerability but are not taken into consideration. This can make defining the MTD, and consequently RP2D, challenging. Alternative methods of determining tolerable doses and regimens are, therefore, urgently needed.

There is increasing interest from clinicians, industry, and regulators, including the US Food and Drug Administration (FDA) and the European Medicines Agency (EMA), to enhance the accuracy of toxicity reporting and incorporate the patient's experience in the drug development process through the use of patient‐reported outcomes (PROs).^8^, ^9^, ^10^

PROs are defined as measurements of a patient's health status that come directly from the patient.^11^ PROs have been extensively studied in the clinical trial and routine care settings and are reliable, feasible and valued by clinicians and patients.^12^, ^13^, ^14^, ^15^, ^16^, ^17^, ^18^, ^19^, ^20^ PROs, in conjunction to CTCAE data, could provide different yet complementary data to inform a more complete understanding of a drug's tolerability and support the selection of tolerable doses and schedules.^21^ PRO collection in early phase trials may become more crucial as drugs developed for specific molecular subtypes with small patient populations may receive regulatory approval on the basis of early phase trials resulting in PROs not being collected in later‐phase trials. A recent example is the FDA approval of the neutrotrophic tyrosine receptor kinase (NTRK) inhibitor entrectinib in patients with NTRK gene fusion solid tumours or ROS1 positive metastatic non‐small‐cell‐lung cancer.^22^

The FDA has taken steps towards the inclusion of the patient perspective in all stages of the drug development process, including issuing guidance on the use of PROs in drug development, and collaborating with industry to form the PRO consortium with the aim of developing robust patient‐reported symptom measurement tools.^11^

Despite the desire to increase PRO use in drug development, there is minimal literature regarding the use of PROs in early phase oncology trials. A review of PRO use across all clinical trials (including non‐oncological trials) from 2007 to 2013 showed phase 1 trials were less likely to include a PRO measure (PROM) compared with phase 2 trials.^23^ A systematic review from 2012 to 2016 identified 15 phase 1 oncology trials with health‐related quality of life (HRQOL) as an endpoint, none of which used HRQOL to inform the RP2D.^24^ However, this study only included published trials, placing it at risk of publication bias. PRO inclusion is also a relatively recent phenomena, so examining published trials alone may reflect choices made by trial investigators from several years prior. Trials in progress may have higher rates of PRO inclusion due to increasing awareness of PROs among early phase trialists.

The feasibility and importance of assessing PROs was highlighted in a single‐centre study in which 80 PRO‐CTCAE items were assessed in phase 1 oncology patients at baseline, mid‐cycle 1 and 2.^25^, ^26^, ^27^ Completion rates were high (98.7%). Significant under‐reporting of certain AEs by clinicians was noted, including fatigue, anxiety, and pain. There was also poor patient‐clinician agreement on sexual health, cognition, and urination. This emphasised the potential role of PROs in complementing clinician‐assessed CTCAE gradings to formulate a more comprehensive understanding of a drug's tolerability. PRO‐CTCAE responses, particularly interference, have clinically relevant consequences and correspond with DLTs, dose interruption, and drug discontinuation. This provides further evidence of their usefulness in assessing drug tolerability.^28^

We conducted a database search of ClinicalTrials.gov to describe characteristics and trends of PRO use and reporting in early phase oncology trials. ClinicalTrials.gov is the largest global trial registry and a valuable resource for assessing the trends and characteristics of registered clinical trials.^29^ By including trials that are completed and in progress, this will provide a more current and complete assessment of PRO use.

## 2 METHOD

### 2.1 Study design and search strategy

This cross‐sectional study included early phase dose‐finding oncology trials (DFOT) with start dates 01/01/2007–20/01/2020 registered on ClinicalTrials.gov. Ethics approval was not required for this database review of ClinicalTrials.gov. Data were extracted in XML format on 1 February 2020. DFOT were identified using the following search strategy in the advanced search function: condition or disease – cancer; study type – interventional (clinical trial); study results – all trials; recruitment status – not yet recruiting, recruiting, enrolling by invitation, active not recruiting, suspended and completed; expanded access – available, no longer available, temporarily not available, approved for marketing; age: child, adult, older adult; sex – all; study phase – early phase 1, phase 1; funder type – NIH, other US federal agency, industry, all others; date restriction (01/01/2007–20/01/2020). The search was then repeated to identify DFOT with a PRO endpoint by adding outcome measures – ‘patient‐reported outcome’ OR ‘Health‐related quality of life’ OR ‘quality of life’ OR ‘QOL’ OR ‘PRO’ OR ‘PROMS’ OR ‘HRQOL’.

The ClinicalTrials.gov database entry for each search result was reviewed to confirm inclusion criteria were met. Eligible trials were DFOT (phase 1 or phase 1/2 oncology trial with a dose finding component) assessing an intervention (drug or radiotherapy), which included at least one PRO as an endpoint. Haematology and paediatric trials were included. Trials that did not meet all eligibility criteria were excluded.

### 2.2 Data extraction

Data was extracted using a pre‐defined data abstraction form in Microsoft Excel (Text S1). Study characteristics were extracted: title, study period, sponsor's country of origin, sponsor type, number of participating centres, number of patients enrolled, study phase, dose escalation study design, tumour type, type of therapy undergoing dose escalation, primary endpoint for phase 1, and current study activity status. PRO characteristics were then extracted: number of PROs included, type of PROs, PRO endpoint (primary, secondary, exploratory), phase in trial in which PROs were collected, method of collection, person completing the PRO, frequency of PRO assessment and duration of PRO follow‐up.

#### 2.2.1 Patient‐reported outcome reporting

Data were extracted regarding PRO outcomes and the statistical methods used for PRO analysis from two sources:

1. Completed trials with PRO results available on the ClinicalTrials.gov database
2. Trials with published manuscripts listed on the ClinicalTrials.gov database.

There are no guidelines regarding how PRO data should be reported in non‐randomised trials. The following details were extracted based on the CONSORT‐PRO checklist for randomised trials^30^: whether PRO results were published, whether PRO results were described in the abstract, whether a PRO hypothesis was stated, whether relevant PRO domains were identified, the content validity of the PRO, timing of PRO assessments, statistical methods for PRO analysis, presence of missing data and reasons for missing data, handling of missing data, whether a minimal clinically important difference (MCID) was described and whether PRO data informed the MTD or RP2D.

All data were checked for internal consistency and disagreements resolved by discussion among the investigators.

### 2.3 Data analysis

Data were analysed using R version 3.6.1. For continuous variables, summary statistics of median and range were displayed. For categorical variables, frequencies (number) and percentages were displayed. Linear regression models with ‘year of study initiation’ as the independent variable were fitted to assess the trend of the number and percentage of trials, and number of sponsoring countries with PRO endpoints over time.

## 3 RESULTS

**FIGURE 1:**
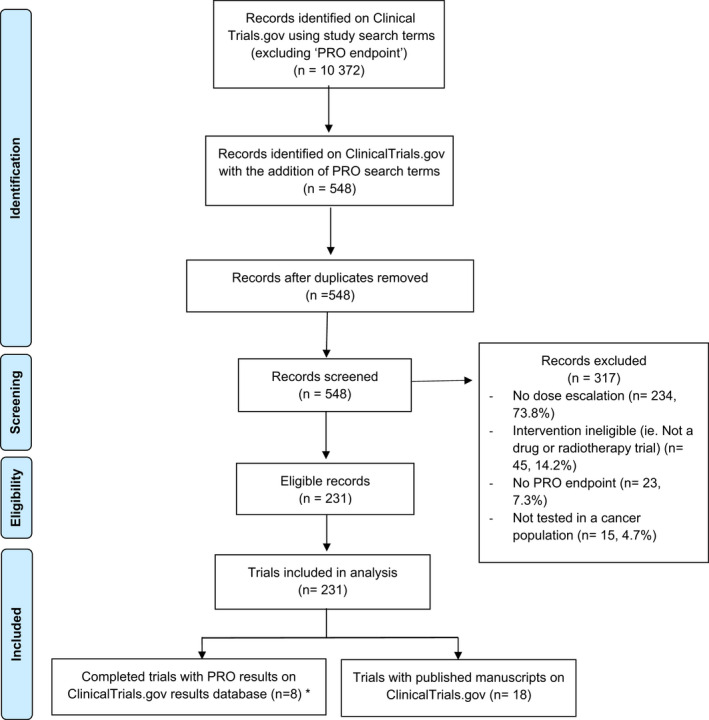
PRISMA diagram. * 3 trials had published manuscripts on ClinicalTrials.gov

A total of 10 372 DFOTs were identified on ClinicalTrials.gov. In total, 548 (5.3%) DFOTs included a PRO endpoint, and 231 (42.2%) met eligibility criteria (Figure 1): adult (224, 97%), paediatric (7, 3%), solid tumour (175, 75.8%) and haematology (56, 24.2%) (Table 1). Phase 1/2 (108, 46.8%) trials were most common, followed by phase 1 dose escalation (101, 43.7%) and phase 1 dose escalation and expansion trials (22, 9.5%).

**TABLE 1:** All eligible trials – study characteristics (*n* = 231)

	Number (%)
Study population	
Adult	224 (97.0%)
Paediatric	7 (3.0%)
Cancer	
Solid tumour	175 (75.8%)
Haematology	56 (24.2%)
Phase	
Phase 1 dose esc	101 (43.7%)
Phase 1 dose esc and exp	22 (9.5%)
Phase 1 and 2	108 (46.8%)
Anti‐cancer agent	
Drug combinations	119 (51.5%)
Single agents	110 (47.6%)
Not described	2 (0.9%)
Escalating treatment in dose escalation	
Targeted therapy	94 (40.7%)
Immunotherapy	33 (14.3%)
Radiotherapy	33 (14.3%)
Chemotherapy	32 (13.9%)
Complementary/alternative	20 (8.7%)
Vaccine	6 (2.6%)
Radionuclide	5 (2.2%)
Other	5 (2.2%)
Hormonal	2 (0.9%)
Antibiotic	1 (0.4%)
Primary endpoint	
MTD	107 (34.9%)
Safety	95 (30.9%)
DLT	57 (18.6%)
RP2D	26 (8.5%)
Response rate	16 (5.2%)
Feasibility	2 (0.7%)
HRQOL	2 (0.7%)
Recurrence‐free survival	1 (0.3%)
Missing	1 (0.3%)
Sponsor type	
All others (individuals, universities, organisations)	135 (58.4%)
Industry	83 (35.9%)
US NIH	13 (5.6%)
Number of centres	
1	108 (46.8%)
2–5	56 (24.2%)
6–10	20 (8.7%)
>10	46 (19.9%)
Not specified	1 (0.4%)
Study design	
Other	151 (65.4%)
3+3 dose escalation	64 (27.7%)
Continual reassessment method	12 (5.2%)
Accelerated titration	3 (1.3%)
Rolling 6	1 (0.4%)
Study activity	
Recruiting	114 (49.4%)
Completed	77 (33.3%)
Active, not recruiting	33 (14.3%)
Not yet recruiting	6 (2.6%)
Suspended	1 (0.4%)
PRO endpoint	
Primary	4 (1.7%)
Secondary	207 (89.6%)
Exploratory	18 (7.8%)
Primary and secondary	2 (0.9%)
PRO phase	
Dose escalation only	115 (49.8%)
Phase 1 and 2	54 (23.4%)
Dose escalation and expansion	31 (13.4%)
Phase 2	28 (12.1%)
Dose expansion only	3 (1.3%)
Number of PRO measures included	
1	137 (59.3%)
2	67 (29.0%)
3	17 (7.4%)
4	5 (2.2%)
5	4 (1.7%)
7	1 (0.4%)
PRO measure (*N* = 119, top 10 listed)	
EORTC QLQ C30	78 (21.1%)
Not specified	49 (13.3%)
EQ 5D−5L	19 (5.1%)
Brief Pain Inventory	10 (2.7%)
PRO‐CTCAE	10 (2.7%)
FACT‐prostate	7 (1.9%)
FACT‐General	7 (1.9%)
EORTC QLQ LC13	7 (1.9%)
FACT‐lymphoma	6 (1.6%)
EORTC QLQ Brain‐20	6 (1.6%)
PRO suites	
Others	124 (33.6%)
EORTC	119 (32.2%)
FACT	52 (14.1%)
Not specified	49 (13.3%)
PROMIS	11 (3.0%)
MDASI	8 (2.2%)
Peds QOL	6 (1.6%)
Frequency assessment	
Unknown	140 (60.6%)
Other	87 (37.7%)
Monthly	2 (0.9%)
Weekly	2 (0.9%)
Method collection	
Unknown	230 (99.6%)
Electronic	1 (0.4%)
Person completing PRO	
Not stated	218 (94.4%)
Patient	12 (5.2%)
Patient or carer	1 (0.4%)

Abbreviations: MTD, maximum tolerated dose; DLT, dose‐limiting toxicity; RP2D, Phase 2 recommended dose; PRO, patient‐reported outcome; QoL, quality of life; HRQOL, health‐related quality of life.

### 3.1 All eligible trials

#### 3.1.1 Study characteristics

**FIGURE 2:**
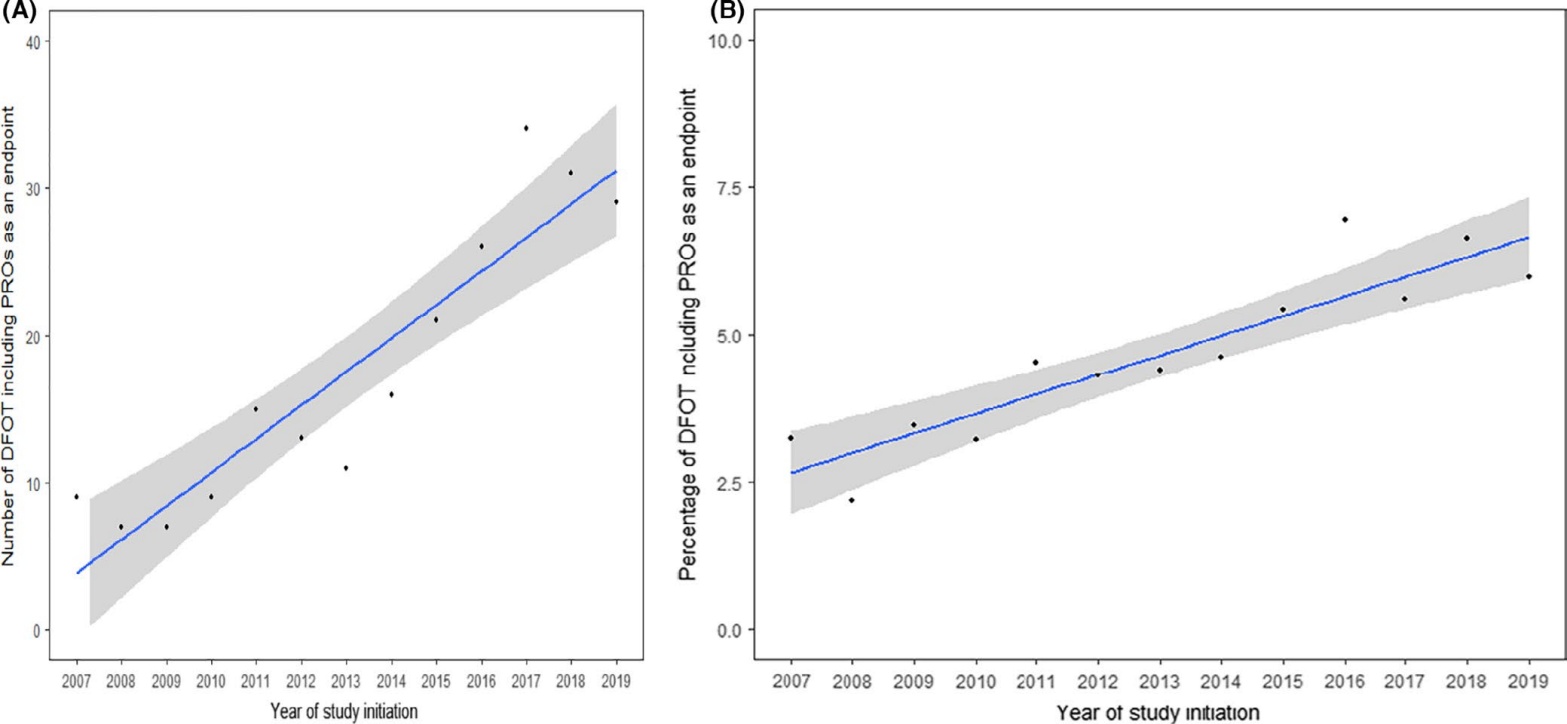
(A) Trends in the number of DFOT using PROs as an endpoint on ClinicalTrials.gov (2007–2019). (B) Trends in the percentage of DFOT using PROs as an endpoint on ClinicalTrials.gov (2007–2019)

**FIGURE 3:**
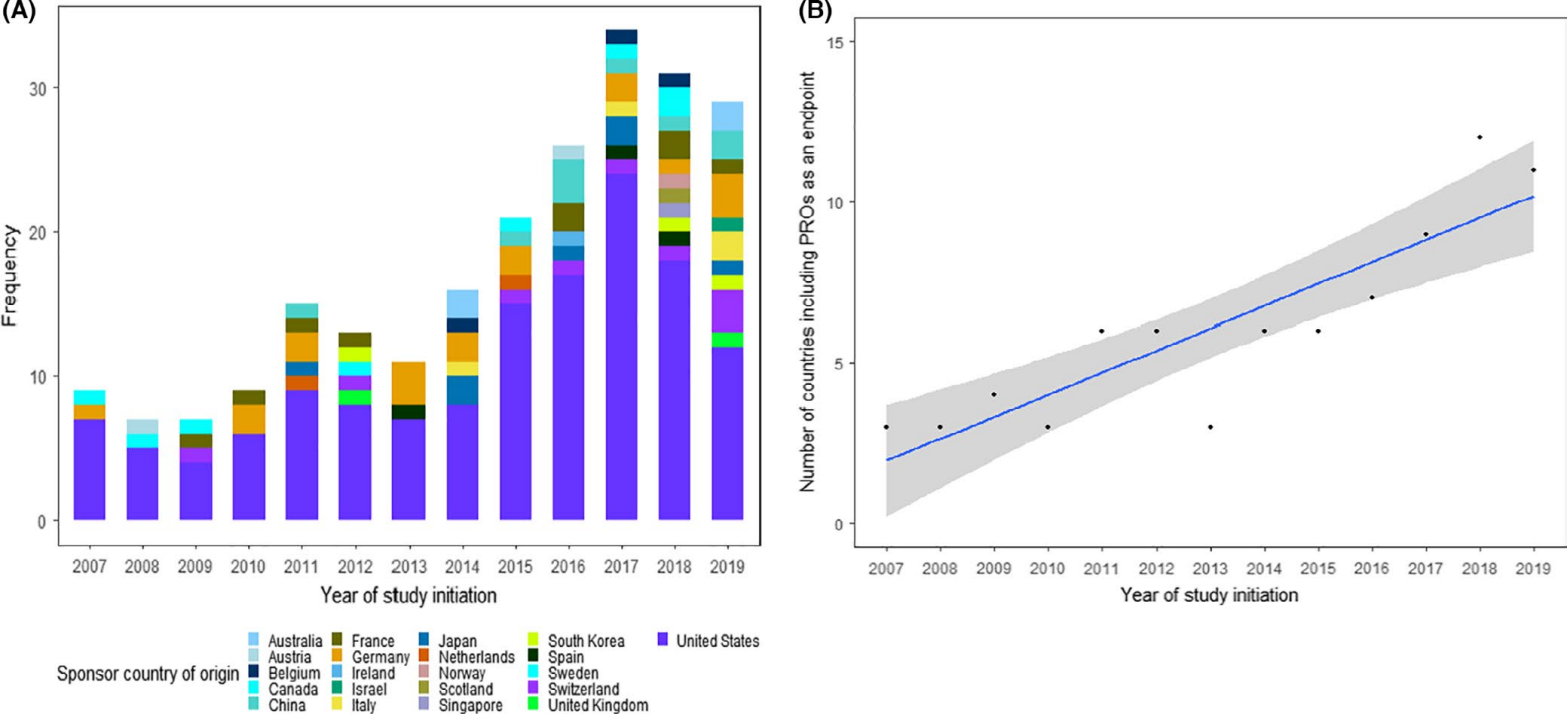
(A) Trends in PRO usage by sponsor country of origin on ClinicalTrials.gov (2007–2019). (B) Trends in the number of sponsoring countries using PROs as an endpoint on ClinicalTrials.gov (2007–2019)

The number and percentage of DFOT with PRO endpoints increased by 2.3 trials per year (95% confidence interval (CI): 1.6–2.9) (Figure 2A) and 0.3% per year (95% CI: 0.2–0.4) (Figure 2B) respectively. There was also an increasing number of countries with institutions sponsoring DFOT with PRO endpoints, from three countries in 2007 to 11 in 2019 (Figure 3A), at a rate of 0.7 countries per year (95% CI: 0.4–0.9) (Figure 3B).

Trials were predominantly conducted in adults (224, 97.0%) with solid tumours (175, 75.8%), sponsored by individuals, universities or other organisations (135, 58.4%). A similar number involved drug combinations (119, 51.5%) or single agents (110, 47.6%). The most common therapies in escalation were targeted therapy (94, 40.7%), immunotherapy (33, 14.3%) and radiotherapy (33, 14.3%). Most trials did not specify their study design (151, 65.4%). 64 (27.7%) used a ‘3+3’ dose escalation design and 12 (5.2%) used a continual reassessment method. MTD (107, 34.9%) and safety (95, 30.9%) were the most common primary endpoints. A total of 114 (49.4%) were still recruiting, and 77 (33.3%) were completed.

#### 3.1.2 Patient‐reported outcome characteristics

PROs were typically secondary endpoints (207, 89.6%). Most trials (137, 59.3%) used 1 PROM (range: 1–7). PROs were most frequently collected in the dose escalation (115, 49.8%) and phase 1 and 2 (54, 23.4%). A total of 119 unique PROMs were used. The most common were the EORTC QLQ C30 (78, 21.1%), EQ‐5D‐5L (19, 5.1%), the Brief Pain Inventory (10, 2.7%) and PRO‐CTCAE (10, 2.7%). Information on the frequency of PRO assessment was missing in 140 (60.6%) trials, and the method of PRO collection was missing in 230 (99.6%) trials. Only 13 trials (5.6%) specified the person completing the PRO.

### 3.2 Eligible trials with reported results on ClinicalTrials.gov

Fifteen completed trials had results available on ClinicalTrials.gov database, of which eight reported their PRO results specifically (Figure 1). The study characteristics and PRO reporting characteristics are presented in Table S1. Seven trials presented descriptive statistics, and one presented least‐square means of treatment effects from a linear mixed effects (LME) model.

### 3.3 Eligible trials with published manuscripts listed on ClinicalTrials.gov

#### 3.3.1 Study characteristics

Eighteen trials had published manuscripts listed on the ClinicalTrials.gov database (Table 2). Trials were primarily sponsored by individuals, universities or other organisations (9, 50.0%). Most used a ‘3+3’ dose escalation design (10, 55.6%). The treatments most common in dose escalation were targeted therapy (7, 38.9%) and chemotherapy (6, 33.3%). All trials except one had safety and/or MTD as their primary endpoint.^31^

**TABLE 2:** Published trials with manuscripts available on ClinicalTrials.gov – study characteristics (*n* = 18)

Study	Sponsor country of origin	Sponsor type	Study design	Population	Treatment in dose escalation	Dose escalation design	Primary endpoint
Taylor et al.^32^, ^33^ (NCT00692900)	United States	Individuals, universities or other organisations	Phase 1 dose escalation	Adults, recurrent ovarian/ primary peritoneal/ fallopian tube cancer	Chemotherapy – intraperitoneal oxaliplatin^32^ or intraperitoneal docetaxel^33^	3+3	MTD, DLT
Wyatt et al.^34^ (NCT01585246)	United States	Individuals, universities or other organisations	Phase 1/2	Adults, prostate cancer	Complementary therapy – Saw Palmetto	Continual reassessment method	MTD, feasibility, efficacy
Van Zandwijk^35^ (NCT02369198)	Australia	Industry	Phase 1 dose escalation	Adults, mesothelioma or metastatic non–small cell lung cancer	Targeted therapy – Epidermal Growth Factor Receptor –targeted, EnGeneIC Delivery vehicle packaged, miR‐16 mimic (TargomiRs)	3+3	MTD, DLT, efficacy
Haddad et al.^36^ (NCT01276496)	United States	US National Institute of Health	Phase 1 dose escalation	Adult, advanced solid tumours	Targeted therapy – Cilengitide	3+3	MTD, safety
Sampath et al.^37^ (NCT01923506)	United States	Individuals, universities or other organisations	Phase 1 dose escalation	Adults, prostate cancer	Radiotherapy – Stereotactic radiotherapy	Modified rolling 6	MTD
Reiss et al.^38^ (NCT01264432)	United States	US National Institute of Health	Phase 1 dose escalation	Adults, peritoneal carcinomatosis	Targeted therapy – veliparib	3+3	MTD
Dinkic et al.^39^ (NCT01238770)	Germany	Individuals, universities or other organisations	Phase 1 dose escalation	Adults, recurrent ovarian cancer	Targeted therapy – pazopanib	6 patients to be treated at each dose level or less if 2 dose limiting toxicities (DLTs) developed at 1 dose level	MTD
Laetsch et al.^31^ (NCT02637687)	Germany	Industry	Phase 1 dose escalation	Paediatric, central nervous system tumours	Targeted therapy‐ LOXO‐101	Modified rolling 6	Safety
Aguilar et al.^40^ (NCT00638612)	United States	Industry	Phase 1 dose escalation	Adults, pancreas	Immunotherapy‐ Gene‐mediated cytotoxic immunotherapy	Not specified	Safety
Subbiah et al.^41^ (NCT02240238)	United States	Industry	Phase 1/2	Adult, advanced solid tumours	Chemotherapy‐ Nanoparticle cisplatin	Continual reassessment method	MTD, RP2D
Crew et al.^42^ (NCT00516243)	United States	US National Institute of Health	Phase 1 dose escalation	Adult, breast cancer	Complementary therapy‐ Polyphenon E	Time to event continual reassessment method	MTD
Watanabe et al.^49^ (NCT01763788)	United States	Individuals, universities or other organisations	Phase 1/2	Adult, squamous non‐small cell lung cancer	Chemotherapy‐ gemcitabine	3+3	MTD
Kumar et al.^43^ (NCT01864018)	United States	Individuals, universities or other organisations	Phase 1/2	Adult, multiple myeloma/ light chain amyloidosis	Chemotherapy‐ cyclophosphamide	3+3	MTD
Verstovsek et al.^44^ (NCT00509899)	United States	Industry	Phase 1/2	Adult, primary myelofibrosis and polycythaemia vera/ essential thrombocythemia	Targeted therapy‐ JAK2 inhibitor INCB018424	3+3	Safety, MTD
Boulin et al.^46^ (NCT01040559)	France	Individuals, universities or other organisations	Phase 1 dose escalation	Adult, hepatocellular carcinoma	Chemotherapy‐ idarubicin loaded microspheres	Modified continual assessment method	MTD, safety
Verstovsek et al.^45^ (NCT01423851)	Japan	Individuals, universities or other organisations	Phase 1 dose escalation	Adult, primary myelofibrosis, post polycythaemia vera myelofibrosis or post essential thrombocythemia myelofibrosis	Targeted therapy‐ NS‐018 (JAK 2 inhibitor)	3+3	Safety, MTD
Siegel et al.^47^ (NCT01129570)	United States	Individuals, universities or other organisations	Phase 1 dose escalation	Adult, hepatocellular carcinoma	Complementary therapy – Siliphos (milk thistle)	3+3	MTD
Martin‐Broto et al.^48^ (NCT02275286)	Italy	Individuals, universities or other organisations	Phase 1/2	Adult, sarcoma	Chemotherapy – trabectidin	3+3	DLT, RP2D

Abbreviations: DLT: dose‐limiting toxicity; MTD: maximum tolerated dose; RP2D: Phase 2 recommended dose.

Three trials also listed their PRO results on the ClinicalTrials.gov database. As expected, more detailed descriptive and inferential analyses were provided in the manuscripts compared with the entries on the ClinicalTrials.gov database.

#### 3.3.2 PRO reporting

One trial (NCT00692900) conducted the dose escalation in two parallel arms and reported each arm separately in two publications.^32^, ^33^ This resulted in 19 publications from 18 trials (Table 3).^31^, ^32^, ^33^, ^34^, ^35^, ^36^, ^37^, ^38^, ^39^, ^40^, ^41^, ^42^, ^43^, ^44^, ^45^, ^46^, ^47^, ^48^, ^49^ Only one trial included PROs as a co‐primary endpoint.^34^ No trials stated a PRO hypothesis. All trials used a valid PRO instrument. The most commonly used PROMs were the EORTC QLQ C30 (7, 26.9%), FACT‐hepatobiliary (2, 7.7%), International Prostate Symptom Score (2, 7.7%) and the Myelofibrosis Symptom Assessment Form (2, 7.7%). The majority collected PROs during the Phase I dose escalation (11, 61.1%). Most (16, 88.9%) collected PROs at baseline and subsequent time points. No trials identified who was responsible for completing the PROs or how PROs were collected.

**TABLE 3:** Published trials with manuscripts available on ClinicalTrials.gov – PRO characteristics (*n* = 18)

Study	PRO result in abstract	PRO instrument	Phase	Frequency of assessment	Statistical methods	How were the PRO outcomes analysed?	MCID described?	Missing PRO data present?	Number of patients with missing PRO data described?	Reasons for missing PRO data described?	Method for managing missing PRO data described?	PRO considered for MTD/RP2D?
Taylor et al.^32^, ^33^ (NCT00692900)	Yes	MDASI with ovarian cancer specific items	Phase 1 dose escalation	Weekly	Descriptive analysis	Over dose Over time (baseline to each time period)	No	Yes	Yes	Partial	No	Used to confirm the MTD
Wyatt et al.^34^ (NCT01585246)	Yes	IPSS, FACT‐Prostate	Phase 1/2	IPSS: baseline, weeks 2–10, 12, 14 and 22. FACT‐P: baseline, week 12, 14 and 22	Descriptive analysis and inferential statistic	Baseline differences between treatment groups [two‐sample *t*‐test] Over time and between treatment groups [LME]	No	Not described	No	No	Yes	No
Van Zandwijk^35^ (NCT02369198)	No	EORTC QLQ C30	Phase 1 dose escalation	Weekly	Descriptive analysis	Over time(from baseline to 8 treatment weeks)	No	Yes	Yes	No	No	No
Haddad et al.^36^ (NCT01276496)	No	PRO‐CTCAE	Phase 1 dose escalation	Weekly	Descriptive analysis	Not described	No	Yes	Yes	Yes	No	No
Sampath et al.^37^ (NCT01923506)	Yes	IPSS, SHIM, Merrick rectal function scale	Phase 1 dose escalation	Baseline and at each follow‐up appointment for 3 years	Descriptive analysis and Inferential statistic	Over dose and over time, and between patient subgroups [statistical test not stated]	No	Yes	No	No	No	No
Reiss et al.^38^ (NCT01264432)	Yes	EORTC QLQ C30	Phase 1 dose escalation	Baseline, every 2 cycles, during follow‐up	Inferential statistic	Over time [paired *t*‐test]; Over different patient subgroups [two‐sample *t*‐test]	Yes	Yes	Yes	Yes	Yes	Used to confirm the MTD
Dinkic et al.^39^ (NCT01238770)	No	EORTC QLQ C30, EORTC Ovar‐28	Phase 1 dose escalation	Baseline, every 3 cycles, during follow‐up	Unclear	Over time [statistical test not stated]	No	Not described	No	No	No	No
Laetsch et al.^31^ (NCT02637687)	No	Peds QL Infant, Peds QL Core module, Wong Baker Pain Faces	Phase 1 dose escalation	Every cycle	Descriptive analysis	Over time (baseline to each time period)	Yes	Yes	Yes	No	No	No
Aguilar et al.^40^ (NCT00638612)	Yes	FACT‐hepatobiliary	Phase 1 dose escalation	Baseline, follow up	Descriptive analysis	Over time (baseline to each time period) and over different treatment arms	No	Yes	Yes	No	No	No
Subbiah et al.^41^ (NCT02240238)	Yes	EORTC QLQ C30	Phase 1/2	Not stated	Descriptive analysis	Over time (baseline to last assessment)	No	Yes	Yes	No	No	No
Crew et al.^42^ (NCT00516243)	No	SF‐36	Phase 1 dose escalation	Baseline, 6 months	Unclear	Over time [statistical test not stated]	No	Not described	No	No	No	No
Watanabe et al.^49^ (NCT01763788)	No	LCSS, ASBI	Phase 1/2	Not stated	Descriptive analysis and Inferential analysis	Time to worsening via Kaplan–Meier estimates and over treatment groups [cox regression]	Yes	Not described	No	No	No	No
Kumar et al.^43^ (NCT01864018)	No	FACT/GOG neurotoxicity questionnaire	Phase 1/2	Baseline, after each cycle for 4 cycles, then every 3 cycles	Descriptive analysis	Over time (for each cycle)	No	Yes	Yes	No	No	No
Verstovsek et al.^44^ (NCT00509899)	No	Myelofibrosis Symptom Assessment Form, EORTC QLQ C30	Phase 1/2	Baseline, 1 and 6 months	Descriptive analysis and Inferential statistic	Over dose, over time and over treatment groups [statistical test not stated]	No	No	No	No	No	No
Boulin et al.^46^ (NCT01040559)	No	EORTC QLQ C30	Phase 1 dose escalation	Baseline, D15, D30 and D60 post TACE	Descriptive analysis	Over dose and over time (baseline to 1 month at each dose) [Mean difference and 95% CI stated]	Yes	Yes	Yes	No	No	Used to confirm the MTD
Verstovsek et al.^45^ (NCT01423851)	Yes	Myelofibrosis Symptom Assessment Form	Phase 1/2	Baseline, Day 1 of Cycles 2 and 4, Day 1 of every 3 cycles thereafter	Descriptive analysis	Over time (after 4 and 12 weeks of treatment)	No	Yes	No	No	No	No
Siegel et al.^47^ (NCT01129570)	No	FACT‐hepatobiliary	Phase 1 dose escalation	Week 1, 6, and 12	Unclear	Only baseline data was reported	No	Not applicable	No	No	No	No
Martin‐Broto et al.^48^ (NCT02275286)	No	EORTC QLQ C30	Phase 1/2	Every 3 months for 24 months	Descriptive analysis and Inferential statistic	Over time (baseline to cycle3) [Mann‐Whitney or Kruskal‐Wallis]	No	Not described	No	No	No	No

Abbreviations: ASBI, Average Symptom Burden Index; IPSS, International Prostate Symptom Scale; LCSS, Lung Cancer Symptom Scale; LME, linear mixed effects; MCID, minimal clinically important difference; MDASI, MD Anderson Symptom Inventory; MTD, maximum tolerated dose; RP2D, phase 2 recommended dose; SF‐36, 36‐item Short Form Survey; SHIM, Sexual Health Inventory for Men; TACE, trans‐arterial chemoembolization.

The statistical methods for PRO analysis were variably described. Analysis approaches were classified into three categories: descriptive statistics, inferential statistics (using the observed data to draw inferences, generalise and make judgements about the larger population, usually via hypothesis testing with *p*‐values) or a combination. Nine (50.0%) used descriptive statistics, 1 (5.6%) used inferential statistics, 5 (27.8%) used both. Three (16.7%) did not provide sufficient details on their statistical methods to enable classification. Trials using descriptive analysis described changes in PRO scores over time using different categories, such as those whose PRO scores improved, remained stable or declined.^35^, ^41^, ^44^, ^45^ Among those trials using inferential statistics, most conducted simple statistical tests such as the *t*‐test to assess whether there were significant differences in PRO scores between baseline and after start of treatment, or among different treatment arms. One trial used a Cox regression model to compare time of worsening of symptom scores between two randomised treatment groups in the phase 2 component and presented the estimated hazard ratio and CI. Another trial used an LME model to account for repeated measures over time and allowed for missing data at random. Least‐square means of the treatment effects were obtained from an LME model (with adjustment for baseline PRO scores) to assess whether the experimental treatment had a significant effect on PROs over time compared with placebo in its exploratory randomised controlled component.^34^ Sixteen (88.9%) analysed PRO data over time, four (22.2%) analysed PRO data over different doses. Four (22.2%) trials described an MCID. Nine (50.5%) reported the number of patients with missing data, and two (11.1%) reported the reasons for missing data. Two (11.1%) described their methods for dealing with missing data.

With regards to using PROs to define tolerable doses and regimens, 3 (16.7%) used PROs to confirm the MTD. However, these do not state whether PROs were reviewed prior to determining the MTD or whether the MTD was retrospectively deemed tolerable after PRO data were reviewed.

Seven (39.8%) trials included PRO results in their abstract. All trials reported their PRO results in their primary trial manuscript rather than in a separate manuscript.

## 4 DISCUSSION

This is the first study to examine current trends in PRO use and reporting in DFOT. PRO use in DFOT increased over time and in a wider variety of settings. The trial characteristics are representative of the current drug development landscape, with a trend towards combinations of therapies in seamless phase 1/2 trials with novel dose‐finding statistical designs such as the Continual Reassessment Method.^50^, ^51^, ^52^, ^53^ Around half (58.4%) were sponsored by individuals, universities or other organisations, primarily representing academic institutions. This is nearly double the rate of academic sponsorship of DFOT on ClinicalTrials.gov for the same time period (2628/10372, 25.3%), indicating the key role that academic‐sponsored trials play in driving the inclusion of patient‐centred endpoints.

Nevertheless, overall use remains limited. DFOT including PROs remain a small proportion of all DFOT (Figure 2B), and this only increased by 2.3 trials per year (Figure 2A). PROs were predominantly used as a secondary or exploratory endpoint, indicating that researchers are not using PROs for the primary endpoint of dose determination. Generic cancer or disease‐specific PROMs were mainly used. However, these may not adequately capture the breadth of toxicities a patient may experience on DFOT and may not be fit for the purpose of assessing tolerability. Some trials used item libraries such as the PRO‐CTCAE (10, 2.7%) and EORTC item library (1, 0.3%), which may be useful for examining specific toxicities from novel agents not covered by legacy measures. Further work is needed to determine how best to select these items. A balance will need to be struck between thoroughness (such as including all 78 symptomatic toxicities in the PRO‐CTCAE to ensure unexpected toxicities are not missed) and feasibility (whether patients will complete lengthy PROMs on a weekly basis).^54^ A possible compromise^55^ is to collect a core set of items from an item library representing common and clinically relevant treatment‐related symptoms^56^ and anticipated toxicities from pre‐clinical trials,^57^ with a free text item to ensure unexpected toxicities are not missed.^58^

The number of trials reporting PRO results was also limited. Only 8 completed trials had PRO results available on the ClinicalTrials.gov database, and only 18 trials published their PRO results in a manuscript listed on ClinicalTrials.gov, indicating a significant amount of research waste. This is also an issue in later‐phase trials with around 20% reporting their PRO results.^59^, ^60^

Although there are no guidelines to inform how PRO results should be reported for non‐randomised trials, the CONSORT‐PRO extension provides a useful checklist of items that should be reported to enable interpretation of PRO results. Key information such as a PRO objective, the person completing the PRO, and the method of PRO collection was not described in any publications. Furthermore, the statistical approach to the PRO data, MCID, the presence of missing data (and the reasons for missing data) were only described in some publications. This limits the interpretation and usefulness of the PRO data for making decisions regarding tolerability. It also limits the capacity to develop hypotheses and inform power calculations for later‐phase trials. This is again consistent with the later‐phase trial setting, where studies have shown that PRO reporting quality is suboptimal.^61^, ^62^, ^63^, ^64^

Although most trials collected PRO data over different doses and over several time points, only 22.2% analysed PRO data over different doses and time. More than 60% reported PRO data over time only. This fails to use the richness of the data to further inform tolerability at different doses. In addition to descriptive summary measures, several regression approaches can be used to analyse PRO data over time and different doses such as LME model or generalised estimating equation framework, which can account for the correlation of PRO measurements at different time points within an individual.^65^, ^66^ Only a small proportion (16.7%) used PROs to confirm the MTD. It is unclear how the PRO information was incorporated to inform the MTD, and whether it was reviewed prior or after the determination of MTD.

The strengths of this study are that by searching ClinicalTrials.gov rather than published trials we generated a current and complete assessment of PRO use. Many DFOT are never published or there can be a significant lag time between completion and publication.^67^, ^68^ Therefore, searching for published trials only may provide a limited picture of PRO use. We included all forms of DFOT, including haematology, paediatric and radiotherapy trials, which have been excluded from prior reviews.^24^ Our search strategy included all trials commencing from 2007 when registration of US‐based trials to ClinicalTrials.gov became compulsory, ensuring this review is as current as possible. However, not all trials sponsored outside the United States are registered on ClinicalTrials.gov, which may result in those trials being under‐represented in our study.

Other limitations include the use of the ClinicalTrials.gov advanced search function, which can return slight variation in the number of search results day‐to‐day. This was mitigated by creating a copy of the database prior to data extraction. Our search strategy did not include specific PROMs, which may have resulted in some DFOT being missed. However, given that 119 unique PROMs were identified using our search strategy, it is likely that the vast majority of DFOT using PROs were captured by using generic QOL‐ and PRO‐related terms. There was also significant variability in the quality of data provided for each trial. We cannot be certain these data are accurate as we did not review the individual trial protocols or published manuscripts. However, the Clinical Trials Transformation Initiative suggests that information is more likely to be complete if the trial is evaluating drugs/devices that are sponsored by US or multinational organisations which the majority of these trials were.^69^ Although inaccuracies and missing data may impact on the quality and characteristics of the trials, the purpose of this study was to examine trends and characteristics in PRO use. This is more likely to introduce random error rather than bias as the issues are likely to be distributed similarly across time.

The evaluation of PRO reporting quality was not performed as a systematic review. There may be trials which have published their results but have not updated their records on ClinicalTrials.gov. However, there were several similarities noted with our study and the systematic review by Fiteni et al.,^24^ including the type of PROMs used and inadequacies in PRO result reporting, reinforcing that our findings are a fair representation of the characteristics and quality of PRO reporting in DFOT.

Further work is necessary to determine the degree of acceptability of PROs to key stakeholders, potential benefits and barriers and the role of PRO data in defining tolerable doses and regimens. A qualitative study examined patient and clinician attitudes towards the collection of electronic PROs in early phase oncology trials.^70^ While most agreed PROs could provide a more comprehensive understanding of a drug's toxicity, clinicians expressed concerns about monitoring PRO data and the need for careful decision‐making regarding data flow and symptom attribution. Our group is currently conducting a global survey exploring these questions in clinicians, statisticians, trial managers, funders and regulators.

Adaptation of existing methodological guidelines for the inclusion of PROs in randomised trials (e.g. SPIRIT‐PRO extension, CONSORT‐PRO extension, SISAQOL)^30^, ^71^, ^72^ will be needed to standardise protocol content, reporting and statistical analysis in early phase trials. Guidance from regulators such as the EMA and FDA will also enhance uptake.^11^ Simultaneous publication of PRO results alongside the main trial manuscript should be encouraged to minimise research waste.^73^

## 5 CONCLUSION

PRO use in DFOT has increased over time, and across a wider variety of settings. However, overall use remains limited. Few trials reported their PRO results and the quality of the PRO reporting was highly variable. Further work is needed to understand key stakeholder attitudes towards PROs in DFOT, and the potential benefits and barriers to their inclusion. Consensus is needed as how to best integrate PRO data into dose‐finding trials. If PROs are to be included in a meaningful way, adaptation of methodological guidelines for protocol content, statistical analysis and reporting will be required to standardise and improve the quality of PRO data in dose‐finding oncology trials.

## ETHICS APPROVAL AND CONSENT TO PARTICIPATE

Not applicable.

## CONFLICTS OF INTEREST

AM has served on advisory boards and received fees from Merck, FARON, Novartis, Bayer and Janssen, which are all unrelated to this work. CY has received fees from FARON and honorarium from Celgene, which are all unrelated to this work. JLK and ZY declare no conflicts of interest.

## AUTHOR CONTRIBUTIONS

JLK, CY – study design, review of ClinicalTrials.gov, analysis of results, manuscript preparation, approval of final manuscript; ZY– analysis of results, manuscript preparation, approval of final manuscript; AM – study design, analysis of results, manuscript preparation, approval of final manuscript.

## Supporting information

Table S1

Text S1

## Data Availability

Data are available on request from the corresponding author.
