# Supplementary material for: Trends in patient‐reported outcome use in early phase dose‐finding oncology trials – an analysis of ClinicalTrials.gov
Source: Cancer Med. 2021 Oct 22;10(22):7943–57. doi: 10.1002/cam4.4307 (PMC8607259; doi:10.1002/cam4.4307)
Supplement: Supplementary file 1 — Table S1 [file CAM4-10-7943-s001.docx]

**Supplementary Table 1: Completed trials with PRO results available on the ClinicalTrials.gov database (n=8)**

| **Study** | **Sponsor country of origin** | **Sponsor type** | **Study design** | **Population** | **Treatment in dose escalation** | **Dose escalation statistical design** | **Primary endpoint** | **PRO instrument** | **Phase** | **Frequency of assessment** | **Statistical methods** | **How were the PRO outcomes analysed?** |
| --- | --- | --- | --- | --- | --- | --- | --- | --- | --- | --- | --- | --- |
| Wyatt et al ^34^  (NCT01585246) | United States | Individuals, universities or other organisations | Phase 1/2 | Adult, prostate cancer | Complementary therapy- Saw Palmetto | Continual reassessment method | MTD, feasibility, efficacy | IPSS, FACT-Prostate | Phase 1/2 | HRQOL: Baseline, week 12, 14, & 22. IPSS: Baseline, week 3-12, 14, & 22. | Linear mixed effects model | The longitudinal comparisons of IPSS, FACT-P between the Saw Palmetto and placebo group was presented. Each value was created as an average over time from a linear mixed effects model that adjusted for baseline values. |
| Phase I Multicenter, Open-label, Clinical and Pharmacokinetic Study of Lurbinectedin (PM01183) in Combination With Weekly Paclitaxel, With or Without Bevacizumab, in Patients With Selected Advanced Solid Tumors  (NCT01831089) | Spain | Industry | Phase 1 dose escalation | Adult, breast, ovarian, gynaecological, head and neck, non-small cell lung cancer, small cell lung cancer | Chemotherapy and targeted therapy- PM01183 and paclitaxel | Not specified | RP2D, MTD | EORTC QLQ C15-PAL | Phase 1 dose escalation | Through study completion | Descriptive analysis (Mean (SD) reported) | Mean (SD) for individual symptoms, emotional functioning, overall QOL over time and in different treatment groups were reported |
| A Phase I/II Study of the ALK Inhibitor CH5424802/ RO5424802 in Patients With ALK-Rearranged Non-Small Cell Lung Cancer Previously Treated With Crizotinib  (NCT01871805) | United States | Industry | Phase 1/2 | Adult, ALK+ metastatic non-small cell lung cancer | Targeted therapy- CH5424802/ RO5424802 | Not specified | RP2D, DLT | EORTC QLQ C30, EORTC QLQ C13 | Phase 2 | Baseline, Weeks 6, 9, 12, 15, 18, 21, 24, 27, 30, 33, 36, 39, 42, 45, 48, 51, 54, 57, 60, 63, 66, 69, 72, 75, 78, 81, 84, 87, 90, 93, 96, 99, 105, 111, 117, last visit (up to 194 weeks) | Descriptive analysis (Mean (SD) reported) | Mean (SD) for each time point, each subscale and individual symptoms were reported. |
| A Phase 1/Randomized Phase 2 Study to Evaluate LY2603618 in Combination With Pemetrexed and Cisplatin in Patients With Stage IV Non-small Cell Lung Cancer  (NCT01139775) | United States | Industry | Phase 1/2 | Adults, metastatic non-small cell lung cancer | Targeted therapy- LY2603618 | Not specified | RP2D | LCSS, ASBI | Phase 2 | Randomization to the end of study (approximately 12 months after the last participant entered treatment) | Descriptive analysis (Mean (SD) reported) | Mean (SD) presented to show the change from baseline to post-baseline assessment in different treatment groups. |
| Watanabe et al ^49^  (NCT01763788) | United States | Individuals, universities or other organisations | Phase 1/2 | Adult, squamous non-small cell lung cancer | Chemotherapy- gemcitabine | 3+3 | MTD | LCSS, EuroQol-5D-3L | Phase 1/2 | Baseline, Cycle 4 (Cycle = 3 weeks) | Descriptive analysis  (Mean (SD) reported) | Mean (SD) presented to show the change from baseline to post-baseline assessment in different treatment groups. |
| Phase I/Comparative Randomized Phase II Trial of TRC105 Plus Bevacizumab Versus Bevacizumab in Bevacizumab-Naive Patients With Recurrent Glioblastoma Multiforme  (NCT01648348) | United States | United States National Institute of Health | Phase 1/2 | Adult, glioblastoma multiforme | Targeted therapy- TRC105 | 3+3 | MTD | EORTC QLQ C15-PAL, EORTC QLQ BN20, WIWI | Phase 2 | Baseline and 4 weeks | Descriptive analysis, 2-sided t-test and proportion test | **EORTC QLQ C15-PAL and EORTC QLQ BN20:** Mean (range) presented to show the change from baseline to post-baseline assessment in different treatment groups.2-sided t test used to compare the change in scores for single items between different arms.  **WIWI:** Proportion test used to compare the percentage of patients answering yes in different arms. |
| Verstovsek et al^44^  (NCT00509899) | United States | Industry | Phase 1/2 | Adult, primary myelofibrosis and polycythemia vera/ essential thrombocythemia | Targeted therapy- JAK2 inhibitor INCB018424 | 3+3 | Safety, MTD | Myelofibrosis Symptom Assessment Form, EORTC QLQ C30 | Phase 1/2 | Baseline and Week 24 | Descriptive analysis  (Mean (SD) reported) | Mean (SD) presented to show the change from baseline to post-baseline assessment for each dose level. |
| A Phase 1b/2 Study of Repeat rAdiation, Minocycline, and Bevacizumab in Patients With Recurrent gliOma (RAMBO)  (NCT01580969) | United States | Individuals, universities or other organisations | Phase 1/2 | Adult, glioma | Antibiotic- minocycline | Not specified | Safety | MDASI-Brain Tumour | Phase 1/2 | From start of study treatment until 26 weeks after radiation therapy (29-30 weeks) | Descriptive analysis  (Mean (SD) reported) | Means (SD) from baseline and 26 weeks post radiation post radiation among different dose levels were reported. |

Grey shading: trials that also had published manuscripts on ClinicalTrials.gov (Tables 2 and 3)

MTD: Maximum Tolerated Dose; RP2D: Phase II Recommended Dose.

MDASI: MD Anderson Symptom Inventory; IPSS: International Prostate Symptom Scale; LME: linear mixed effects; SHIM: Sexual Health Inventory for Men; SF-36: 36-item Short Form Survey; LCSS: Lung Cancer Symptom Scale; ASBI; Average Symptom Burden Index; WIWI: was it worth it.

SD: standard deviation
