# Supplementary material for: Trends in patient‐reported outcome use in early phase dose‐finding oncology trials – an analysis of ClinicalTrials.gov
Source: Cancer Med. 2021 Oct 22;10(22):7943–57. doi: 10.1002/cam4.4307 (PMC8607259; doi:10.1002/cam4.4307)
Supplement: Supplementary file 2 — Text S1 [file CAM4-10-7943-s002.docx]

**SUPPLEMENTARY TEXT 1**

1. **Data extracted from ClinicalTrials.gov**

- Study characteristics
  - Study title
  - Study period (year of study initiation, year of study conclusion)
  - Sponsor country of origin
  - Sponsor type (academic, industry, US NIH, US federal agency)
  - Number of participating centres (1, 2-5, 6-10, >10)
  - Number of patients enrolled (actual or predicted)
  - Study phase (phase 1 dose escalation, phase 1 dose escalation and dose expansion, phase 1 and 2)
  - Dose escalation study design (3+3 dose escalation, continual reassessment method, rolling 6, accelerated titration, other)
  - Patient population (adult, paediatric)
  - Tumour type (advanced cancers vs specific tumour type, solid tumour vs haematology)
  - Type of therapy undergoing dose escalation (chemotherapy, immunotherapy, targeted therapy, radiotherapy, complementary/alternative, vaccine, radionuclide, hormonal, antibiotic, other)
  - Primary endpoint for phase 1 component of study (safety, DLT, MTD, RP2D, feasibility, pharmacokinetics, response rate, HRQOL, PRO, recurrence free survival)
  - Current study activity status (not yet recruiting, recruiting, active not recruiting, enrolling by invitation, completed, suspended)
- PRO characteristics:
  - Number of PROs included
  - PRO name(s)
  - Type of PRO (generic, tumour specific, item library, other) and free text
  - PRO endpoint (primary, secondary, tertiary, exploratory)
  - Phase of trial in which PROs were collected (dose escalation, dose expansion, dose escalation and expansion, phase 2, phase 1 and 2)
  - Method of collection (paper, electronic, telephone, unknown)
  - Person completing the PRO (patient, carer, nurse, patient or carer, patient or nurse, not stated)
  - Frequency of assessment (weekly, fortnightly, monthly, other, unknown)
  - Duration of PRO follow up
